# Nanocellulose-based polymer hybrids and their emerging applications in biomedical engineering and water purification

**DOI:** 10.1039/c9ra03261d

**Published:** 2019-06-18

**Authors:** Dinesh K. Patel, Sayan Deb Dutta, Ki-Taek Lim

**Affiliations:** The Institute of Forest Science, Kangwon National University Chuncheon 24341 Republic of Korea; Department of Biosystems Engineering, College of Agriculture and Life Sciences, Kangwon National University Chuncheon 24341 Republic of Korea ktlim@kangwon.ac.kr

## Abstract

Nanocellulose, derived from cellulose hydrolysis, has unique optical and mechanical properties, high surface area, and good biocompatibility. It is frequently used as a reinforcing agent to improve the native properties of materials. The presence of functional groups in its surface enables the alteration of its behavior and its use under different conditions. Nanocellulose is typically used in the form of cellulose nanocrystals (CNCs), cellulose nanofibers (CNFs), or bacterial nanocellulose (BNC). CNCs and CNFs have a high aspect ratio with typical lengths of ∼100–250 nm and 0.1–2 μm, respectively; BNC is nanostructured cellulose produced by bacteria. Nanohybrid materials are a combination of organic or inorganic nanomaterials with macromolecules forming a single composite and typically exhibit superior optical, thermal, and mechanical properties to those of native polymers, owing to the greater interactions between the macromolecule matrix and the nanomaterials. Excellent biocompatibility and biodegradability make nanocellulose an ideal material for applications in biomedicine. Unlike native polymers, nanocellulose-based nanohybrids exhibit a sustained drug release ability, which can be further optimized by changing the content or chemical environment of the nanocellulose, as well as the external stimuli, such as the pH and electric fields. In this review, we describe the process of extraction of nanocellulose from different natural sources; its effects on the structural, morphological, and mechanical properties of polymers; and its various applications.

## Abstract 2

Nanocellulose, derived from cellulose hydrolysis, has unique optical and mechanical properties, high surface area, and good biocompatibility.

## Graphical abstract

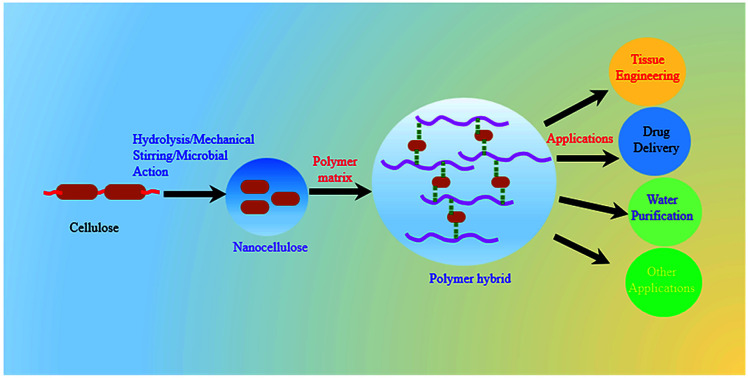

## 1. Introduction

**Fig. 1:**
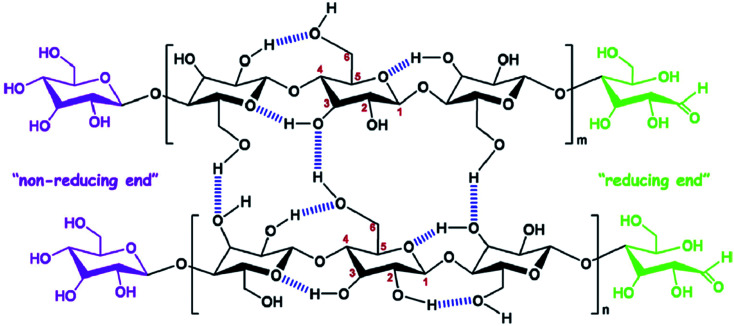
Chemical structure and intra, intermolecular hydrogen bonding in cellulose. Reproduced with permission from ref. 6; Copyright 2014, Elsevier.

Cellulose is the most abundant polymer in nature^1^ and consists of polysaccharides with long chains of β-d-glucopyranose units assembled by β-1,4 glycosidic bonds, which form a dimer known as cellobiose.^2^ It is an important constituent of plant cell walls, where it provides mechanical support; it is also present in other organisms such as bacteria, fungi, algae, and even sea animals.^3^ Cellulose contains both disordered and ordered regions in its polymer chains and, owing to its renewability, low cost, abundance, and biodegradability, is a promising material for biomedical applications.^4^ The polysaccharide chains assemble to form microfibrils, which, in turn, assemble to form macro fibers and fibers, through numerous hydrogen bonds.^5^ The chemical structure and intra- and inter-molecular hydrogen bonds in cellulose are depicted in Fig. 1.^6^ Nanocellulose is a form of nano-structured cellulose, which is typically used in the form of cellulose nanocrystals (CNCs), cellulose nanofibrils (CNFs), or bacterial nanocellulose (BNC). CNCs, also known as cellulose nanowhiskers, are needle-like cellulose crystals with typical widths of 10–20 nm and lengths of 100–250 nm;^7^ they are produced from several biological sources, such as wood, cotton, wheat straw, rice husk, bamboo,^8^ potato tuber, sugar beet, ramie,^6^ bacteria,^9^ and algae*,*^10^ typically through strong acid hydrolysis.^11^ Acid treatments cause the removal of disordered regions from the source materials, forming highly ordered crystalline cellulose structures.^12^ Therefore, CNCs are crystalline in nature and can be used as reinforcing agents for a wide range of applications.^13,14^ CNFs are a different form of nanostructured cellulose, composed of long fiber networks with typical lengths of 0.1–2 μm and diameters approximately equal or larger than those of CNCs.^15^ CNFs contain amorphous cellulose in their structure and, therefore, are not as crystalline as CNCs.^7^ BNC, also known as microbial cellulose, is another form of nanostructured cellulose, which is produced by bacterial actions; It has a typical cross-section diameter of 20–100 nm and a degree of polymerization (DP) of 4000–10 000.^15^ Other forms of nanocellulose are amorphous nanocellulose (ANC) and cellulose nanoyarns (CNY). A comparison of the these different forms of nanostructured cellulose (CNC, CNF, BNC, ANC and CNY) is shown in Table 1.^16^ The fabrication of high-performance polymer materials using nanoparticles or nanofillers has received significant attention in recent years by both academia and industry.^17^ The hybrid material is a combination of an intimate mixture of inorganic components, organic components or both types of component.^18^ Polymer nanohybrids are composed of a combination of organic or inorganic nanomaterials with polymer matrices into a single composite. The overall properties of nanohybrids are highly influenced by the nature and interactions of the nanomaterials within the polymer matrix.^19^ Nanomaterials have attracted considerable attention in the field of material science owing to their excellent physiochemical properties, which are not possessed by their micro and macroscale analogs.^20^ Several nanomaterials, such as nanostructured metals and metal oxides (Ag, Au, ZnO, *etc.*), nanostructured carbon in different forms (fullerenes, CNTs, and graphite), nanozeolites, and nanocellulose are frequently used to improve the properties of native (pure) polymers.^21–25^

**Table 1:** Comparative study of different types of nanocellulose. Table obtained from ref. 16; published by The AIMS Press

Nanocellulose	Typical sources	Approximate dimensions	Advantages
Cellulose nanocrystals (CNCs)	Hardwood, softwood, plants, agricultural residues, bacteria, *etc.*	4–70 nm in width and 100–6000 nm in length	High surface area, excellent mechanical properties, low density and low coefficient of thermal expansion
Cellulose nanofibrils (CNFs)	Hardwood, softwood, plants, agricultural residues, bacteria, *etc.*	20–100 nm in width, and >10 000 nm in length	Low density, high surface area, and good mechanical strength
Bacterial nanocellulose (BNC)	Low molecular weight sugars such as glucose	10–50 nm in width, and >1000 nm in length	Excellent mechanical strength, high purity, and greater stability
Amorphous nanocellulose (ANC)	Cotton, wood pulp	20–120 nm in width, and 50–120 nm in length	High content of functional groups and high sorption ability
Cellulose nanoyarn (CNY)	Cellulose and cellulose derivatives	100–1000 nm in width, and >10 000 nm in length	High surface area, and high blotting ability

**Fig. 2:**
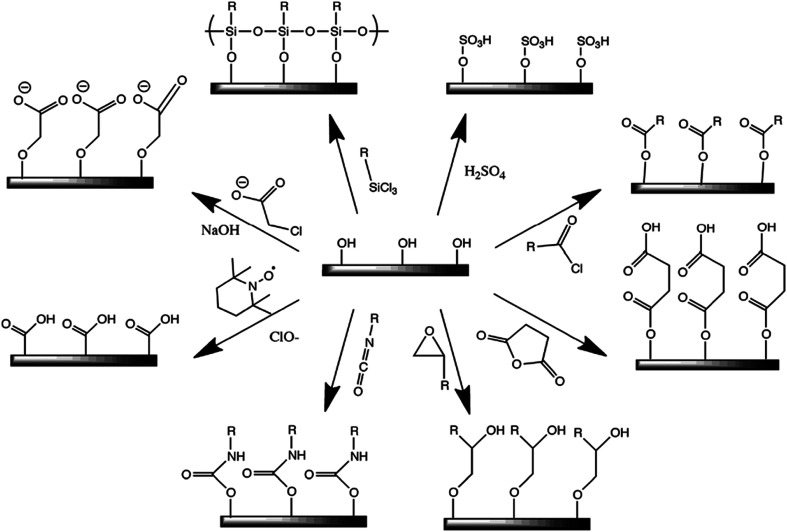
Some common surface modification of nanocellulose: (clockwise from top-right) sulfuric acid treatment provides sulfate esters, carboxylic acid halides create ester linkages, acid anhydrides create ester linkages, epoxides create ether linkages, isocyanates create urethane linkages, TEMPO mediated hypochlorite oxidation creates carboxylic acids, halogenated acetic acids create carboxymethyl surfaces, and chlorosilanes create an oligomeric silylated layer. Reproduced with permission from ref. 29; published by The Royal Society of Chemistry.

Owing to its large surface area (150–250 m^2^ g^−1^), high tensile strength (7.5–7.7 GPa) with Young's moduli of 110–220 GPa,^26^ excellent optical properties, eco-friendly nature, low density, and biodegradability, nanocellulose has recently gained immense attention in the field of nanohybrids for the development of bio-based nanomaterials, such as polymer nanohybrids, mechanically adaptive materials, and mesoporous photonics solids.^27,28^ The presence of hydroxyl groups in the nanocellulose surface provides a platform for chemical functionalization reactions, such as oxidation, etherification, esterification, silylation, and polymer grafting, enabling the nanocellulose to disperse more effectively in various types of polymer matrix materials.^29^ Thus, nanocellulose can be used as a nanofiller.^3,30^Fig. 2 shows a few possible methods for the chemical functionalization of nanocellulose structures.^29^ Recently, polymer nanohybrids based on nanocellulose as a filler material have drawn significant attention in the field of materials science and tissue engineering owing to their versatile nature. Nanohybrids exhibit significantly superior mechanical and optical properties to those of pure polymers.^31^ Furthermore, nanohybrids with nanocellulose as the filler material show higher biocompatibility than their native polymers.^32^

Herein, we have described an approach to isolate nanocellulose from different natural sources, its influence on the polymer behavior, and its functional applications. The effects of the nanocellulose content on the structural, morphological, mechanical, and optical properties and biocompatibility have been investigated in detail. Nanocellulose-based nanohybrids have great potential in biomedical applications such as tissue engineering and wound dressing. Further, the removal of toxic ions from polluted media through nanocellulose-based hybrids membranes represent a new, fascinating strategy for water purification.

## 2. Extraction of nanocellulose

Several steps including washing and drying, grinding, alkali treatment, delignification, and acid hydrolysis are involved in the extraction of nanocellulose from raw biomass. These treatments cause the removal of other non-cellulosic components, such as lignin, hemicellulose, and wax, surrounding the cellulose structure. Furthermore, the acid treatment causes the dissociation of amorphous zones, leading to the generation of crystalline CNCs. On the other hand, the mechanical disintegration of cellulosic materials forms a web-like structure with amorphous and crystalline parts known as CNFs.^33^ Nanocellulose can also be obtained through microbial actions in the form of BNC. It is important to note that chemical constituent of all these nanostructured cellulose types are similar but their solid-state and physical properties are significantly different.^34^ Commercially available microcrystalline cellulose (MCC) and other non-wood plants, *e.g.*, jute, cotton, leaves, and rice husk, are also used for nanocellulose extraction.^35–38^ To obtain the nanostructured cellulose from cellulose, it is necessary to break down the hierarchical structure of cellulose in at least one dimension in the nanometer scale.^39^ This pretreatment requires the removal of the amorphous regions. This includes the grinding and an alkali treatment (NaOH 4%, 80 °C) of the raw material followed by a bleaching process with oxidizing agents such as NaClO_2_, in order to remove the lignin and hemicellulose from the matrix.^40^ The acid hydrolysis and mechanical disintegration of the pretreated material lead to the formation of CNCs and CNFs, respectively.^29,41,42^ The properties of the obtained CNCs are highly influenced by the hydrolysis duration and the nature and concentration of the acid.^3^ It is important to note that hydrolysis with sulfuric acid is preferred to that with other acids because it produces charge moiety on the nanostructured cellulose, which forms a more stable suspension. In addition, the steam explosion process (14–16 bar at 200–270 °C) of raw materials for a short time (20 s to 20 min) has also been used as a pretreatment, to deconstruct the hierarchical structure of the cellulose. This method is more effective for hardwood cellulosic materials than for softwood ones.^40^ Nanocellulose can also be obtained from glucose through the action of bacteria such as *Acetobacter xylinus* and *Acetobacter hansenii*.^43^ For this process, bacteria are grown in a medium with proper carbon and nitrogen concentrations for several days at 30 °C.^44^ The purification of the obtained BNC is carried out by washing it thoroughly with an alkaline solution and water. The obtained BNC are dimensionally uniform and homogeneous.^3^ Several efforts have been made for economical production of BNC through different approach such as fermentation systems, genetic engineering, evaluation of culture media compositions, and post-production modification. However, the technological production of BNC is still extremely expensive and large efforts need to enhance BNC from food to a new generation sophisticated materials for different applications such as pourable and spoonable dressings, cultured dairy products, wound care dressing and in burn therapy, *etc.*^45^

**Fig. 3:**
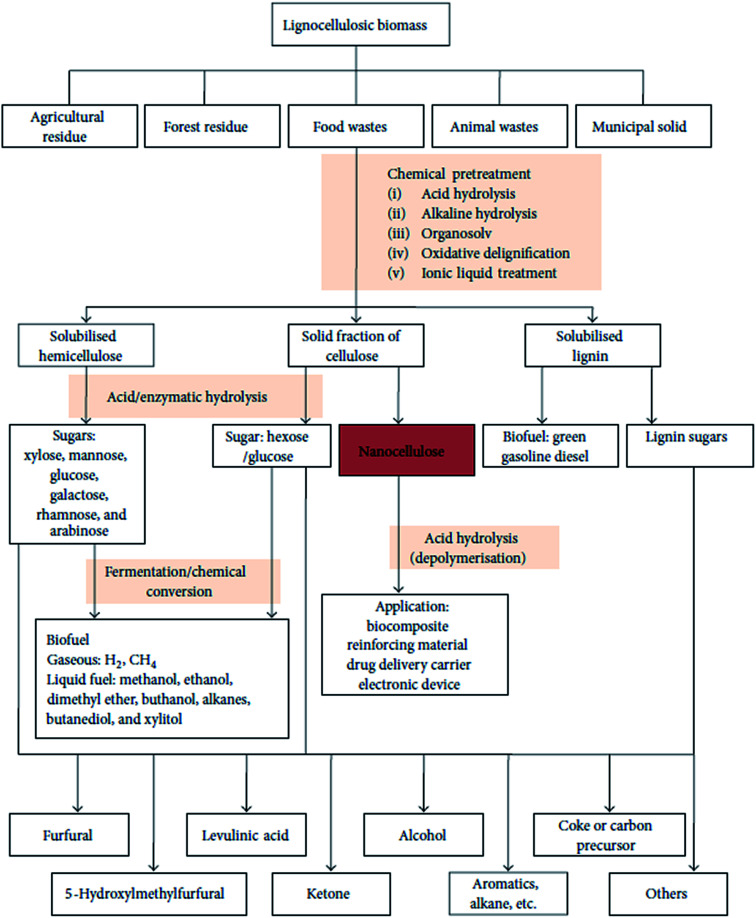
Schematic representation of biomass bio-refinery to nanocellulose intermediate and chemicals. Figure obtained from ref. 46; published by The Hindawi Publisher.

**Fig. 4:**
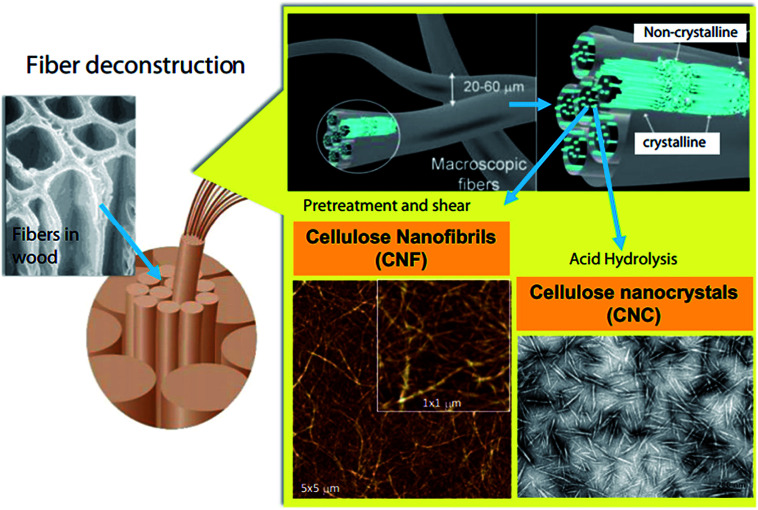
Schematic illustration of CNF and CNC production from fiber cell walls by mechanical and chemical treatments, respectively. Reproduced with permission from ref. 30; Copyright 2014, Elsevier.

A schematic representation of the process of extraction of nanocellulose and other chemicals from raw biomass is shown in Fig. 3; this includes the pretreatment of raw materials for the isolation of cellulose from non-cellulosic materials and further treatments for specific applications.^46^ A schematic representation of the process of CNF and CNC extraction from fiber cell walls through mechanical and chemical treatments are shown in Fig. 4.^30^

## 3. Synthesis of polymer nanohybrids

Nanocellulose is often used as a filler material to enhance the native properties of polymers. Several methods have been used for the synthesis of polymer nanohybrids; including solution casting, melt extrusion, and *in situ* polymerization. In this section, we describe this these methods, along with their advantages and disadvantages.

### 3.1. Solution casting technique

This is the most common method used for the synthesis of polymer nanohybrids. In this process, the filler is dispersed in a suitable solvent through sonication or mechanical stirring followed by the dissolution of the polymer in the same solvent. Then, the solutions are mixed at or above room temperature. Nanohybrid films are obtained by either casting the solution or precipitation.^47^

### 3.2. *In situ* technique

Here, the fillers are dispersed in a suitable solvent at the early stage of polymerization, and curing agents or chain extenders are added for polymerization at a suitable temperature. This technique provides a homogenous dispersion of the filler in the polymer matrix, thereby improving the nanohybrid properties significantly through stronger interactions.^47^

### 3.3. Melt blending technique

Here, an extruder is required to generate high shear forces at high temperatures.^48^ The nanomaterial is incorporated into the molten polymer matrix, which is sheared at a high rate. The most significant advantage of this technique is that no additional solvents are required. However, degradation of the polymer chain may occur due to the high temperature and shear forces.^49^

## 4. Effects of nanocellulose on polymer properties

The interactions between the polymer matrix and the functional groups in the nanocellulose have a strong influence on the overall properties of the developed nanohybrids. This interaction depends mainly on the distribution of the nanocellulose in the matrix. Weak interfacial interactions between the non-polar polymer matrix and the hydrophilic nanocellulose have been previously reported.^30^ This drawback is addressed by surface functionalization of nanocellulose or incorporation of compatibilizers.^50^ In the rest of this section we describe the effects of the nanostructured cellulose on various properties of the polymer, including the structural, morphological, mechanical, thermal, and optical properties by considering a few common examples. These properties determine the potential applications of the developed material and, therefore, need to be evaluated for each developed nanohybrid.

### 4.1. Structural and morphological properties of nanohybrids

**Fig. 5:**
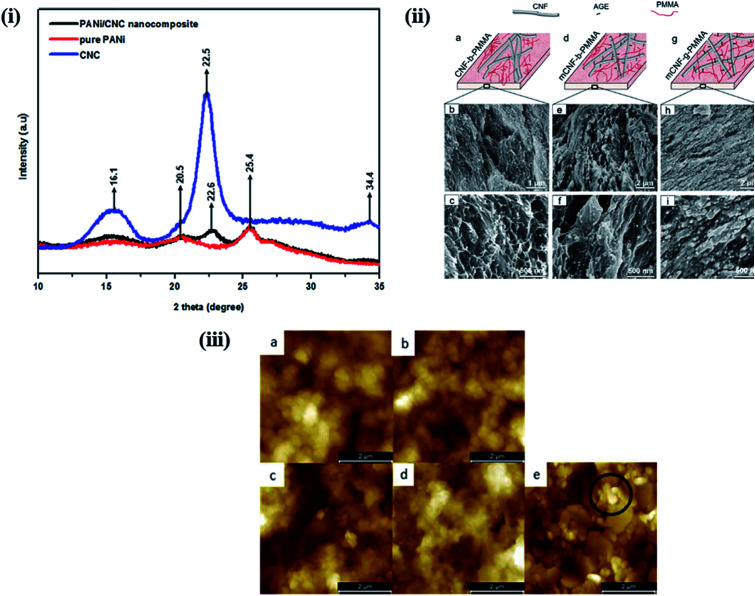
(i) XRD-pattern of nanocellulose, pure PANi and PANi/nanocellulose hybrid. Reproduced with permission from ref. 54; published by The Royal Society of Chemistry, (ii) surface morphology of nanocellulose based PMMA hybrids. (a, d and g) Schematic representation of the relative CNF/PMMA distribution and SEM images of cryo-fractured surface of (b and c) CNF blended with PMMA (CNF-*b*-PMMA), (e and f) modified CNF blended with PMMA (mCNF-*b*-PMMA), and (h and i) modified CNF grafted with PMMA (mCNF-*g*-PMMA). Reproduced with permission from ref. 59; Copyright 2018, American Chemical Society, and (iii) 5 μm × 5 μm AFM images of (a) 5 (z-scale: 800 nm) and (b) 10 (z-scale: 600 nm) layers of CNC-PANI ink and (c) 5 and (d) 10 layers (z-scale: 1000 nm) of the PANI ink printed on MLCC paper and (e) the bare MLCC paper substrate (z-scale: 600 nm). Reproduced with permission from ref. 64; published by The Royal Society of Chemistry.

The properties of nanohybrids are highly influenced by the nature and dispersion of the filler in the polymer matrix. Khan *et al.* reported a CNC-induced trans-crystallization in chitosan polymers, which led to the improvement of the barrier properties of the nanohybrids.^51^ However, an increase in the amorphous nature was observed in polymer-grafted magnetic nanocellulose hybrids, which might be responsible for the enhancement in their hydrophilicity and adsorption capacity.^52^ Similar observations were also reported by Anirudhan *et al.* in nanocellulose/nanobentonite composites attached with multi-carboxyl functional groups as adsorbents for the removal of cobalt(ii).^53^Fig. 5i shows the XRD patterns of PANi-nanocellulose nanohybrids synthesized by *in situ* oxidative polymerization of aniline on the surface of CNCs. It was observed that the crystalline nature of nanocellulose was suppressed by the addition of the polymer. Furthermore, the crystallinity of nanohybrids can be enhanced by increasing the content of CNC in the polymer matrix.^54^ A comparative study was performed by Abraham *et al.* to evaluate the effects of nanocellulose in non-cross-linked and cross-linked natural rubber composites. They observed that nanocellulose agglomeration was higher in the latter and influenced the overall properties of the developed materials.^55^ However, no significant change in crystallinity was observed in poly(3-hydroxybutyrate) (PHB)-based bionanocomposites synthesized *via* solution casting using CNC as a filler material.^56^

The morphological behavior of nanohybrids is highly affected by the compatibility of the filler with the polymer matrix and the interactions between the polymer and the filler material. Nair *et al.* successfully prepared cross-linked and non-cross-linked hydrogels using two different CNF sources with poly(methyl vinyl ether-*co*-maleic acid) and polyethylene glycol. In SEM images of the fractured surfaces of the cross-linked samples, they observed a rougher morphology than in those of the non-cross-linked samples. This is because the cross-linked network structure causes the samples to be stretched more. In addition, non-cross-linked samples showed a relatively open structure with a different pore size, much greater than those in the cross-linked samples.^57,58^ The topographical behavior of the nanohybrids is also influenced by the duration of the filler incorporation in the polymer matrix process during polymerization. Boujemaoui *et al.* reported the morphological differences between two nanohybrids utilizing CNF as the filler in a poly(methyl methacrylate) (PMMA) matrix. By analyzing SEM images, they observed that grafted (mCNF-*g*-PMMA) nanohybrids exhibit a more compact layered structure than the blended (mCNF-*b*-PMMA) nanohybrids; this is because the CNF network was embedded within the methyl methacrylate (MMA) monomers prior to polymerization. The SEM images of the cryo-fractured surfaces of the grafted and blended nanohybrids are shown in Fig. 5ii.^59^ The *in situ* polymerization of poly(vinyl acetate) using vinyl acetate monomers in the presence of CNC yields a more homogenous and ductile fracture surface compared to that of physically mixed hybrids.^60^ A notable improvement in the coating properties was observed in composites synthesized using TEMPO-functionalized CNF with zirconium alkoxide and an epoxy-functionalized silane. The SEM images indicated a decrease in the roughness from composite coating compared to that of the native materials; the roughness further decreased as the content of CNF in the matrix increased. This is due to the strong interaction between the polar groups in the matrix and the functionalized CNF.^61^ Zhang *et al.* studied the influence of the vinyl monomer concentration on the nanocomposites. A rod-like surface morphology with a core–shell structure was observed in PANi-CNC hybrids when the aniline monomer concentration was low during the chemical oxidative polymerization process on the surface of the CNC.^62^ Using atomic force microscopy, Fernandes *et al.* studied the surface topography of chitosan/BNC nanohybrids; they observed a granular morphology of pure chitosan, whereas the nanohybrids displayed randomly assembled BNC.^63^Fig. 5iii shows the surface morphology of a multilayer curtain-coated (MLCC) paper printed using conducting ink based on polyaniline and CNC hybrids synthesized by emulsion polymerization using a dodecylbenzenesulfonic acid (DBSA). The layers printed with pure PANi ink exhibit a more dispersed globular microstructure with a considerably higher roughness than the layers printed with the PANi-CNC ink.^64^ In this section, we have discussed the effects of nanostructured cellulose on polymers in terms of their crystallinity and surface morphology by considering different examples. It was interesting to note that the properties of the developed nanohybrids are highly dependent on the interactions between the polymer matrix and the nanostructured cellulose. Better interactions through homogenous dispersion lead to a significant improvement in the crystallinity through the nucleating effect of nanostructured cellulose. Smoother surface morphologies, attributed to stronger interactions, were observed in nanohybrids compared to those of pure polymers. More examples of the applications of nanocellulose-based polymer hybrids in biomedicine and water purification will be given in the Applications of nanocellulose-based hybrids section.

### 4.2. Mechanical properties of nanohybrids

**Fig. 6:**
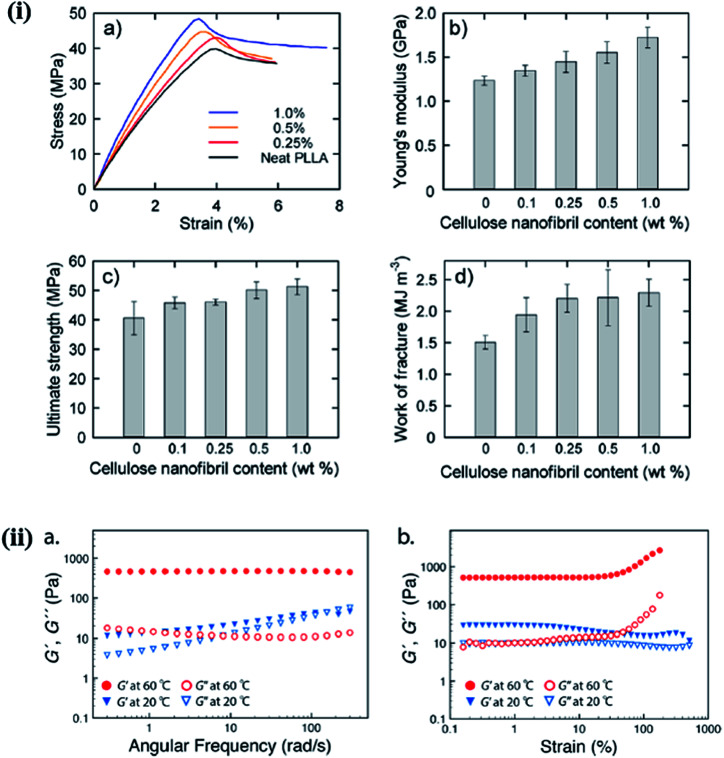
(i) Mechanical properties of nanocomposite films with different cellulose nanofibril contents: (a) stress–strain curves, (b) Young's moduli, (c) ultimate tensile strengths, and (d) work of fractures. Reproduced with permission from ref. 66; Copyright 2013, American Chemical Society, and (ii) typical oscillatory rheological behavior, as shown by the nanohybrid hydrogel with a 1.5 wt% cellulose nanocrystal (CNC) and 1.0 wt% methyl cellulose (MC) loading: (a) frequency sweep preformed between 0.1–300 rad s^−1^ at 60 °C (red) and 20 °C (blue), determined at 10% strain; (b) strain sweeps at 60 °C (red) and 20 °C (blue), determined at an angular frequency of 6.283 rad s^−1^. The linear low strain *G*′ value was *ca.* 510 Pa at 60 °C. Reproduced with permission from ref. 75; Copyright 2014, American Chemical Society.

The mechanical behavior of nanohybrids is significantly influenced by the volume fraction of the filler and its aspect ratio, orientation, and stress transfer, as well as the filler–matrix interactions. In general, the mechanical behavior of nanohybrids has been studied in terms of the content and surface modification of the fillers, and in relation to the coupling materials used.^65^ A significant improvement in the mechanical properties was observed in PEG-grafted CNF and poly(l-lactide) (PLLA) composite over the native PLLA, due to the reinforcement effect of CNF in the polymer matrix and the inhibition of crack propagation. The mechanical behavior of composite films with different CNF contents is depicted in Fig. 6i.^66^ Lee *et al.* studied the mechanical response of CNC/poly(vinyl alcohol) (PVOH) hybrids and observed that the CNC enhanced the crystallinity and alignment of PVOH by acting as a direct reinforcement, thereby improving the stiffness and strength of the nanohybrids. A large enhancement in strength (880 MPa) and stiffness (29.9) was observed when loading 40 wt% of CNC in the polymer matrix.^67^ The Young's modulus is highly affected by the nature of the filler, such as the aspect ratio, whereas the tensile strength is dependent on the matrix behavior. For CNC/carboxymethylcellulose (CMC) hybrids, an improvement in mechanical responses, including the stiffness and strength, was observed due to efficient stress transfer from the polymer matrix to the CNC through strong hydrogen bonding.^68^ A comparative study of the mechanical behavior of nanohybrids was performed by Huan *et al.* to evaluate the reinforcement effect of CNC in electrospun poly(lactic acid) (PLA) fibrous hybrids for two different filler orientations (aligned and random). They observed a significant enhancement in the tensile strength and Young's modulus at a low concentration of CNC (10 wt%), and a deterioration of the same properties at higher concentrations; this is attributed to more efficient stress transfer from the PLA to the stiffer (at a lower concentrations) nanocellulose;^69^ on the other hand, at higher concentrations, the CNC agglomerate, resulting in a decrease in the crystallinity. This behavior is similar for both random and aligned filler orientations; however, in all cases, the improvement in the mechanical properties was much better with the aligned filler compared to the randomly oriented one.^70^ The incorporation of 5% (w/w) CNC in the chitosan matrix resulted in a 26% increase in the mechanical properties, compared to the native polymer.^51^ Starch/CNF-based foam nanohybrids at lower concentrations of CNF (40 wt%) also exhibited an improvement in the mechanical properties, which deteriorated with increasing CNF content. The physical and mechanical responses of CNF-based foam composites with varying contents of CNF are listed in Table 2.^71^ In another study, it was observed that the addition of 4% lignin-coated CNC in an acrylonitrile butadiene styrene (ABS) matrix resulted in an increase in the tensile and storage moduli. Furthermore, a deterioration in mechanical properties was observed at a higher content of the filler, due to the poor interfacial interaction between the polymer and CNC.^72^ The mechanical strength of the hybrid under melt/liquid conditions is governed by several important factors, such as the distribution and dispersion of the filler and its compatibility with the polymer matrix and interfacial interactions. Castro *et al.* studied the mechanical response of poly(vinyl alcohol) (PVA)/BNC hybrids synthesized *in situ* and noted that nanohybrids have a higher storage modulus (*G*′) than their native polymers. This was attributed to the reinforcement effect of BNC *via* hydrogen bonding with the polymer matrix, which is also responsible for the improved mechanical response of the nanohybrids. The reinforcing effect of nanomaterials is highly dependent upon the effective stress transfer between the polymer matrix and the filler material.^73^ Ago *et al.* investigated the effect of CNC on the mechanical properties of lignin-PVA/CNC-based composite under melt conditions. They observed that as the content of CNC increased with a fixed ratio of the lignin-PVA matrix, the storage modulus (*G*′) increased. This is due to the presence of a strong interconnected network structure in the nanohybrids.^74^ McKee *et al.* studied the influence of CNC on the mechanical properties of thermoresponsive methylcellulose (MC) hydrogels under melt conditions, as shown in Fig. 6ii. They noted that by increasing the content of CNC from 0 to 3.5 wt%, the storage modulus was enhanced; in contrast, a distinct gel state was achieved at 60 °C. Their obtained values of the storage modulus (*G*′) were higher than those of the loss modulus (*G*′′) due to the physical cross-links between the methylcellulose and CNC.^75^ These examples clearly indicate that the mechanical properties of nanohybrids are highly influenced by the nature and content of the filler, and the interactions between the nanostructured cellulose and the polymer matrix. These results indicate that nanostructured cellulose can act as a reinforcing agent at a low concentration in solid and at melt conditions.

**Table 2:** Physical and mechanical properties of MFC reinforced amylopectin-based foam with varying MFC contents. The samples have been conditioned in 50% relative humidity and 22 °C for 48 h. The values within parenthesis are the sample standard deviation. Reproduced with permission from ref. 71; Copyright 2008, Wiley Publication

MFC (wt%)	Young's modulus (MPa)	Yield strength (kPa)	Density ρ* (kg m^−3^)	Relative density, (ρ*/*ρ*_s_)^a^	Water content (%)
0	4.9 (1.1)	170 (25)	103 (2.08)	0.084	11.0
10	5.0 (1.0)	310 (91)	109 (2.79)	0.088	10.3
40	7.0 (0.61)	510 (21)	95.1 (1.02)	0.073	8.4
70	1.7 (0.40)	110 (78)	86.5 (1.29)	0.063	7.3

a The theoretical density of the cell wall.

### 4.3. Thermal properties of nanohybrids

**Fig. 7:**
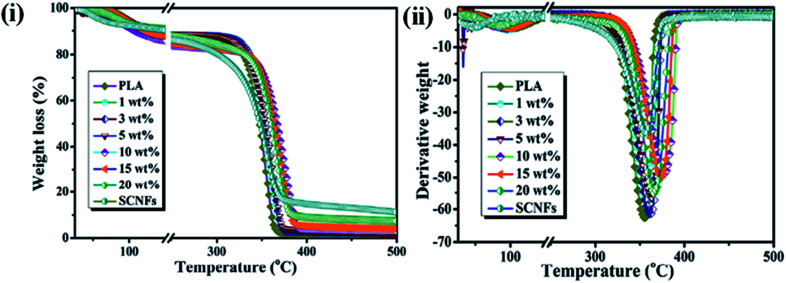
(i) TGA, and (ii) DTA curve of PLA and its indicated nanohybrids. Reproduced with permission from ref. 80; published by The Royal Society of Chemistry.

The thermal stability is another important parameter for hybrid materials; it indicates the temperature range at which materials are stable and is measured by thermogravimetric analysis (TGA). The dispersion of the filler material in a polymer matrix, surface functionalization or adhesion of the filler, and thermal stabilities are the important criteria for the development of reinforced hybrids.^65^ Razalli *et al.* synthesized polyaniline/CNC hybrids through the *in situ* oxidative polymerization of aniline in the presence of CNC isolated from Semantan bamboo and observed an improvement in the thermal stability of the nanohybrids compared to that for pure PANi and CNC, indicating a greater interaction between the matrix and CNC and the protection of the PANi layered by the CNC.^54^ Similar types of degradation behaviors were also observed by Casado *et al.* in PANi/CNF hybrids.^76^ In another study, Gwon *et al.* synthesized poly(lactic acid) (PLA)-based CNC composites using two different CNC, namely pure CNCs and toluene diisocyanate-modified CNC, and evaluated their thermal stabilities by TGA measurements. They observed that the composites made by modified CNC exhibited lower thermal stability than pure PLA and the PLA/CNC composite. This is due to the homogenous dispersion of the functionalized CNC in the polymer matrix, which enhanced the heat transfer rate in the PLA film, leading to faster degradation.^77^ However, an enhancement of thermal stability in the presence of CNC in the PLA matrix was reported by Lu *et al.*, who noted that the thermal stability is highly dependent upon the functionalization and preparation procedure of the CNC.^78,79^Fig. 7i and ii presents a thermogram of PLA/CNC hybrids and indicates that a significant improvement in the thermal stability was obtained in nanohybrids due to the more efficient dispersion of the filler and the stronger interactions between the filler and polymer matrix. Thermal analysis results for pure PLA and its nanohybrids at different filler contents are given in Table 3.^80^ Incorporation of BNC into a poly(vinyl alcohol) (PVA) matrix led to an interesting improvement in the thermal stability at higher temperatures compared to that of pure PVA, possibly due to the better dispersion and excellent compatibility between the PVA matrix and the BNC.^73^ This is further supported by differential scanning calorimetry (DSC) measurements, where an improvement in the glass transition temperature (*T*_g_) and melting temperature (*T*_m_) was observed in nanohybrids. This can be attributed to the strong interaction and excellent compatibility between the matrix and filler, causing a decrease in the mobility of the PVA chains at the interface.^81^ The thermal transition and BNC-induced melting behavior measurements of nanohybrids at various filler concentrations are shown in Table 4.^73^ An improvement in the thermal properties of nanohybrids was also reported by Cho *et al.* in poly(vinyl alcohol)/CNC hybrids synthesized using commercial MCC *via* a casting technique. At lower contents of nanocellulose (1 wt% and 3 wt%), no significant improvement in the thermal stability was observed. However, at a higher content of CNC (5 wt%), the nanohybrids exhibited higher thermal stability.^82^ Moreover, in another study, a decrease in the glass transition temperature (*T*_g_) and melting temperature (*T*_m_) was observed in chitosan/nanocellulose hybrids in the presence of glycerol. This decrease was due to an increase in the movement of the chitosan chains and a decrease in the crystallinity.^83^ Mandal *et al.* synthesized linear and cross-linked PVA-based hybrids using bagasse-extracted nanocellulose and evaluated their thermal behavior. They noted that nanohybrids exhibited higher thermal resistance than pure PVA and cross-linked hybrids. This is due to the interlocking of hydroxyl groups of PVA *via* hydrogen bonding and the formation of a cross-linked network structure, which requires a much higher energy to break.^84^ These results highlighted that the thermal stabilities of hybrids are highly affected by the compatibility of nanostructured cellulose with the polymer matrix. The higher compatibility of nanostructured cellulose with the matrix facilitates the improvement in the thermal behaviors of hybrids through stronger interactions. Thermal properties such as the melting, glass transition, and crystalline temperatures are not directly related to the biomedical applications of hybrid materials but provide important information on the best processing conditions for biomedical polymers into implants or devices. In addition, the thermal stability of material provides information on their applications range. More heat resistant and longer-term thermal properties make polymeric membranes suitable candidates for water treatment.

**Table 3:** Thermal analysis parameters for pure PLA, SCNFs and the nanohybrids with various SCNF (spherical nanocellulose formats) content. Reproduced with permission from ref. 80; published by The Royal Society of Chemistry^a^

Samples	*T*_o_ b (°C)	*T*_5%_ b (°C)	*T*_max_ b (°C)	*T*_f_ b (°C)	*E*_a_ (kJ mol^−1^)
PLA	335.7	325.9	354.6	363.9	319.95
1 wt%	336.3	327.0	357.0	369.4	360.13
3 wt%	342.2	332.4	361.6	371.5	380.61
5 wt%	342.6	333.0	363.1	372.3	407.57
10 wt%	353.8	342.2	376.1	390.0	414.77
15 wt%	350.8	338.9	373.5	383.4	395.16
20 wt%	346.6	331.4	366.3	379.2	373.37
SCNFs	323.1	292.2	358.0	368.3	248.3

a *E*_a_ (activation energy).

b *T*_o,_ *T*_5%,_ *T*_max,_ and *T*_f_ were obtained from the TGA curve at a heating rate of 10 °C min^−1^.

**Table 4:** Thermal transitions and crystallinity (a) of matrices and cross-linked nanohybrids reinforced at various BC loadings. Reproduced with permission from ref. 73; published by The Royal Society of Chemistry

Samples	*T*_g_ (°C)	*T*_m_ (°C)	Δ*H* (J g^−1^)	*X*_c_ (%)	*X*_p_ (%)
PVA 10	76.2	224.7	69.7	0.43	0.43
PVA/BC 10(10)	79.7	236.4	68.3	0.42	0.47
PVA 5	69.2	225.3	73.3	0.45	0.45
PVA/BC 20(5)	78.8	230.6	48.1	0.30	0.37
PVA 2.5	63.5	228.1	59.3	0.37	0.37
PVA/BC 30(2.5)	84.2	229.8	43.9	0.23	0.33

a *X*_c_ = Δ*H*_m_/Δ*H*^0^_m_ and *X*_p_ = *X*_c_/*w*; with Δ*H*^0^_m_ = 161.6 J g^−1^.

### 4.4. Optical properties of nanohybrids

**Fig. 8:**
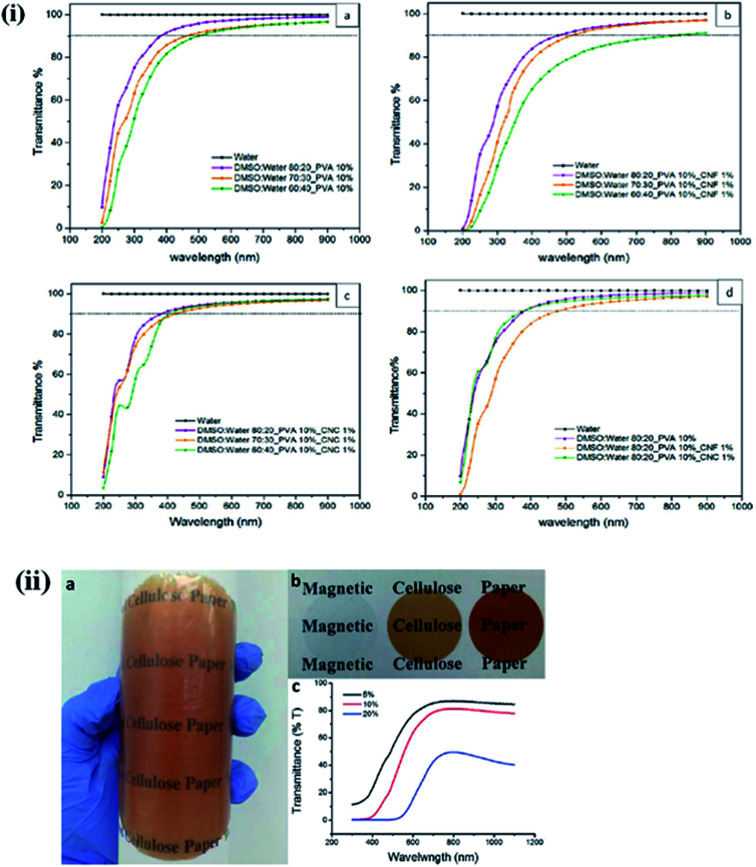
(i) Comparative UV-vis transmission spectrum of 1 mm hydrogels with (a) 10% PVA, (b) 10% PVA and 1% CNF, (c) 10% PVA and 1% CNC, and (d) 10% PVA and 1% CNF/CNC. Reproduced with permission from ref. 31; Copyright 2016, American Chemical Society, and (ii) (a) a digital image of magnetic paper with 10 wt% Fe_3_O_4_ NPs shows the transparency and flexibility, (b) nanopapers with 0 wt%, 5 wt%, and 10 wt% Fe_3_O_4_ NP loading from left to right and (c) transmittance curves of magnetic papers. Reproduced with permission from ref. 96; published by The Royal Society of Chemistry.

The optical transparency is a critical parameter for building light-transmitting materials and solar cell windows and ophthalmic devices. For these applications, materials should be highly transparent. Multifunctional lightweight, transparent, and mechanically strong polymer-based composites are highly desirable for several applications. Geng *et al.* synthesized highly ordered, transparent, and mechanically strong nanohybrids using CNF in a PLA matrix. They observed that chemical grafting of CNF with the PLA matrix resulted in a more aligned and transparent material than physically blended nanohybrids.^85–87^ The influence of the solvent ratio and the type of nanocellulose (CNCs and CNFs) on the optical transparency of poly(vinyl alcohol) hydrogel, was studied by Tummala *et al.* They observed a decrease in the optical transparency of CNFs in the visible range due to the agglomeration of the polymer chains in the media with more aqueous conditions.^88^ On the other hand, nanohybrids having CNCs in their polymer matrix showed a more transparent nature in the visible range than the CNF-based hybrids. This is due to the smaller aspect ratio and lesser light-scattering tendency of the CNC compared to CNFs. Fig. 8i presents the UV-visible transmittance spectrum of PVA hydrogel for different solvent ratios and nanocellulose concentrations.^31^ Lightweight, optically transparent, and mechanically enhanced wood was synthesized by pre-polymerized MMA and a de-lignified porous wood template.^89,90^ The optical properties of the synthesized wood can be altered by changing the cellulose content.^91^ The optical transparency of materials can be tuned using suitable plasticizers.^92,93^ The effect of the ionic crystal, 1-allyl-3-methylimidazolium chloride (Amim Cl), on the optical transparency of CNC was studied by Liu *et al.*, who observed that with an increase in the ionic liquid content during the filtration process, the color changed from light brown to light blue due to the strong interactions between the polar groups of the CNC and the ionic liquid.^94^ In another study, a decrease in the optical transmittance of chitosan/CNF composite was observed when the content of TEMPO-modified CNF in the polymer matrix was increased.^95^ The influence of the CNF on the optical transmittance of uniform and flexible magnetic nanopapers synthesized by immobilization of Fe_3_O_4_ nanoparticles was studied by Li *et al.* They noted that the fabricated nanopapers are transparent in the visible range with excellent flexibility, and that the transparency of the developed magnetic papers can be tuned by changing the content of Fe_3_O_4_ nanoparticles.^96^ A digital image of magnetic papers prepared by using CNF and Fe_3_O_4_ nanoparticles is shown in Fig. 8ii. The transparency of these papers can also be improved by reducing their thickness, which will decrease their optical scattering.^97,98^ Effects of nanocellulose on the polymer properties are also given in Table 5.

**Table 5:** Effects of nanocellulose on the polymer properties

Polymer matrix	Nanocellulose	Preparation technique	Properties	References
Polystyrene (PS)	CNC	Electrospinning	Improvement in tensile strength, wettability, crystallinity, and thermal decomposition	99–101
	CNC	Extrusion	Enhancement in the mechanical properties, degradation temperature	
	CNF-TEMPO	Solution casting	Enhancement in the mechanical, thermal, and optical	
Ethylene vinyl alcohol (EVOH)	CNC	Electrospinning	Enhancement in thermal properties	102 and 103
	BNC			
Poly vinyl chloride (PVC)	CNC	Hot mixing	Enhancement in the mechanical strength	104–106
	CNC	Solution casting		
Polymethyl methacrylate(PMMA)	CNC	Solution casting	Improvement in the mechanical and degradation temperature	107–109
	CNF	Immersion precipitation	Enhancement in thermal stability and storage modulus	
	CNF-TEMPO	Solution casting	Improvement in the mechanical strength	
	CNF	Solvent exchange	Enhancement in mechanical, thermal, and water content	59
Polypropylene (PP)	CNF	Emulsion process	Enhancement in the mechanical strength	110 and 111
	CNC	Melt extrusion	Improvement in the mechanical, thermal and crystallinity	
Poly(lactide) (PLA)	CNC	Solution casting	Enhancement in thermal, mechanical and crystallinity	112 and 113
	CNF			
	CNF-TEMPO		Homogenous dispersion in organic solvents, improvement in mechanical strength, and decrease in the glass transition temperature	66
	CNF	Suspension coating	Enhancement in the mechanical, thermal and crystallization behavior	80
	CNC		Enhancement in the mechanical, water uptake and biocompatibility	114
Polyethylene (PE)	CNC	Melt extrusion	Improvement in thermal and mechanical strength	115
Polyimide (PI)	CNC	Spin coating	Improvement in thermal, mechanical, barrier and optical properties	116
Poly(vinyl alcohol) (PVA)	CNC	Solution casting	Improvement in optical transparency, water content, and mechanical strength	31
	CNF			
	BNC	*In situ* polymerization	Enhancement in thermal, mechanical, and cell proliferation	73
Polyaniline (PANi)	CNC	*In situ* polymerization	Enhancement in electrochemical properties, thermal behavior, and crystallinity	54
	CNC	Emulsion polymerization	Enhancement in electrochemical properties, and mechanical strength	64
Methylcellulose (MC)	CNC	Solution casting	Enhancement in the mechanical strength	75
Poly(butylene adipate-*co*-terephthalate) (PBAT)	CNC	Melt extrusion	Enhancement in thermal, mechanical, and biocompatibility	117
Chitosan	CNF	Solution casting	Enhancement in mechanical, crystallinity and sustained drug delivery	118
Poly(*N*-methacryloyl glycine)	BNC	*In situ* polymerization	Improvement in biocompatibility and sustained drug release	119

## 5. Applications of nanocellulose-based hybrids

### 5.1. Tissue engineering applications

Nanocellulose and its hybrids are frequently used for fabricating a variety of devices, such as medical implants, drug delivery vectors, and vascular grafts; moreover, owing to their sustainability, biodegradability, and biosafety they are used for tissue engineering applications.^120–123^ In addition, high water-holding capacity with better elasticity and conformability and excellent mechanical strength make nanocellulose an ideal material in wound-care dressing applications.^124^ It has been noticed that CNC have antimicrobial activity, which provides an additional advantage for wound-dressing applications.^125^ Notably, more efficient cell adhesion, proliferation, and migration were observed in CNC environments. This is due to the high aspect ratio and good biocompatibility of CNC, which facilitate cellular activities.^6^

**Fig. 9:**
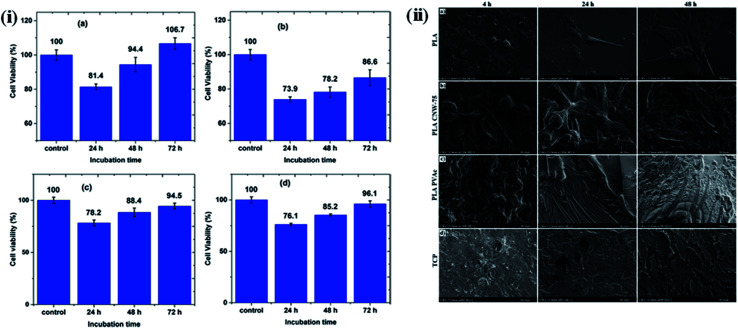
(i) MTT assay cell viability of L929 cells for (a) MA-*g*-PBAT, (b) CNC, (c) 3% CNC, and (d) 9% CNC samples at specified incubation times. Reproduced with permission from ref. 126; Copyright 2017, American Chemical Society, and (ii) influence of materials and surface roughness on NIH-3T3 mouse fibroblast cell morphology and spreading at varying time points (4, 24, and 48 h): (a) control PLA, (b) PLA CNW-75, (c) PLA PVAc, and (d) control TCP (tissue culture plastic). Reproduced with permission from ref. 114; Copyright 2014, American Chemical Society.

Here, we give a few examples of nanocellulose-based polymer hybrids and describe their tissue engineering applications. In these examples, a significant change in cellular responses was observed in the presence of nanocellulose-based hybrids at low concentration, indicating their potential as biomaterials for tissue engineering. The cytotoxic behavior of maleic acid (MA)-grafted poly(butylene adipate-*co*-terephthalate) (MA-*g*-PBAT) hybrids on L929 fibroblasts in the presence of different concentrations of CNCs at different time intervals was studied by Rahimi S K *et al.* using an MTT assay. No notable cytotoxic effects were observed after 72 h of incubation, indicating the biocompatible nature of the developed material. Fig. 9i presents a cell viability test by MTT assay at different time intervals and concentrations of CNC in the hybrids.^126^ In addition, many studies have been conducted on various nanocellulose-based polymer hybrids, such as collagen,^127^ poly(lactic acid),^128^ and polyurethanes,^129^ to evaluate their cytotoxic effects on different cells in a culture media. The effect of CNC on the behavior of a poly(vinyl acetate) (PVAc)-coated poly(lactic acid) (PLA) film in the presence of NIH-3T3 mouse fibroblasts was studied by Hossain *et al.* They observed that the surface of the nanohybrids was completely covered by the fibroblasts, due to their continuous proliferation and spreading, forming a multilayered cellular structure after 24 h of incubation; only a few rounded cells were observed.^114^ The effects of the type of material and surface roughness on the morphology and spreading of NIH-3T3 cells at different time intervals are shown in Fig. 9ii. In another study, Tang J. *et al.* synthesized poly(vinyl alcohol) (PVA)-doped BNC tubular hybrids through a thermally induced phase separation method for artificial blood vessel-based applications. The cytotoxic behavior of the developed hybrids towards pig iliac endothelial cells (PIECs) at different time intervals was monitored using an MTT assay. It was noted that no significant cytotoxic signature was caused by the nanohybrids, and the hybrid tubes enabled a higher proliferation of the cells, indicating their biocompatible nature.^130^ Naseri *et al.* synthesized CNC/sodium alginate and gelatin (SA/G)-based hybrids through a freeze-drying process and evaluated their cytocompatibility using mesenchymal stem cells (MSCs). No sign of cytotoxicity was observed, and the highly porous nature of the scaffold of these hybrids was observed to facilitate cell proliferation.^131^ Poonguzhali *et al.* fabricated chitosan/poly(vinyl pyrrolidone)/nanocellulose (CPN) hybrids through a solution casting technique and evaluated their biocompatible nature using normal mouse embryonic fibroblasts. No cytotoxic effects of the composites were observed; furthermore, the composites with 3% nanocellulose showed a higher level of antibacterial activity than the hybrids with other nanocellulose concentrations.^132^ A wound-healing ability with no inflammation effects was also observed for nanohybrids developed by the *in situ* preparation of silver nanoparticles embedded within a bamboo nanocellulose matrix.^133^

### 5.2. Drug delivery

**Fig. 10:**
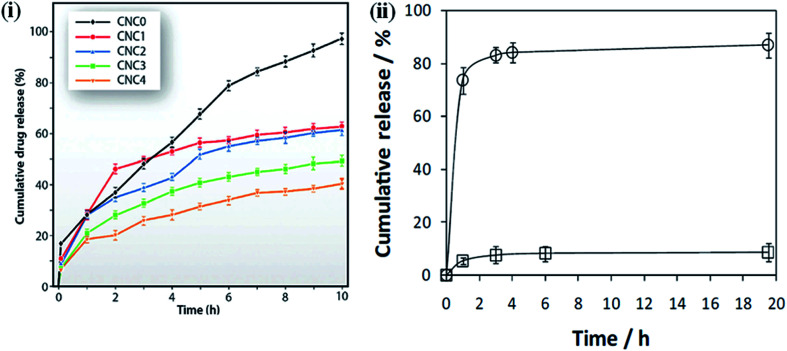
(i) Drug release profiles of polymer and it's indicated nanohybrids. Reproduced with permission from ref. 118; published by The Royal Society of Chemistry, and (ii) diclofenac dissolution profile at pH 2.1 (□) and 7.4 (○) from PMGly/BC/DCF nanocomposite membrane. The lines are for visual guidance only. Reproduced with permission from ref. 119; Copyright 2017, Elsevier.

One of the critical issues of using polymeric materials as implants is that the polymer should release the biologically active bound material at a suitable rate and for the required period of time, which are dependent on the biological conditions.^134,135^ Controlled drug release is a very important factor to maintain the drug concentration within the therapeutic window and achieve the minimum side effects and maximum therapeutic effects of the drug.^136^ Several factors, such as the interaction between the drug and the polymer matrix, the solubility of the drug, and the swelling tendency of films in different media play an important role in the release of drugs from the matrix.^137^ Here, we describe some examples of nanocellulose-based hybrid materials for drug delivery application under different conditions, *i.e.*, the content of nanocellulose, pH, and external electric fields. These examples demonstrated a very controlled release of the loaded drugs in different conditions and show their potential use as carriers in drug delivery applications. The effect of the nanocellulose on the ketorolac tromethamine (KT) drug-delivery behavior of chitosan was studied by Sarkar *et al.* Drug-loaded samples were synthesized by solution casting. The nanohybrids exhibited more sustained drug release compared to the pure chitosan; this behavior was more sustained in nanohybrids with a higher content of nanocellulose. This is due to the greater interaction between the polymer matrix and filler material, which hinders the diffusion pathways and reduces the drug uptake and release.^138,139^Fig. 10i presents an *in vitro* drug release profile of the pure polymer and its nanohybrids.^118^ Shi *et al.* synthesized the BNC/sodium alginate hybrid hydrogels for controlled drug delivery induced by pH and electric stimuli. As the pH of the medium changed from an acidic to an alkaline value, a faster drug release was observed due to the increase in the swelling behavior of the hydrogel in the alkaline medium, causing an easier diffusion of the drug. Moreover, by changing the electric field from 0 to 0.5 V, the drug release from the hybrid hydrogels becomes faster. This is attributed to the swelling nature of the hybrid hydrogels.^140^ Non-cytotoxic and pH-sensitive hybrids were synthesized by Saidi *et al.* using poly(*N*-methacryloyl glycine) and nanocellulose under green-reaction conditions. They observed that faster drug release occurred at a higher pH (7.4) than at a lower pH (2.1). This is due to both the pH-dependent solubility of the loaded drug (diclofenac sodium salt, DCF), which has poor solubility in acidic environments,^141^ and the pH sensitivity of the nanohybrids due to the presence of amino acid properties, which show poorer water–uptake properties under acidic conditions.^119^ An *in vitro* drug release profile of the composites under different conditions is shown in Fig. 10ii. Nanocellulose-based polymer nanohybrids for tissue engineering applications are also summarized in Table 6.

**Table 6:** Nanocellulose-based polymer hybrids for tissue engineering applications

Nanocellulose-based polymer nanohybrids	Nature of materials	Applications	References
BNC/heparin	Scaffolds	Tissue engineering	142–149
BNC/potato Starch (PS)			
BNC/poly(3-hydroxyoctanoate) (PHO)			
BNC/polyvinyl alcohol			
BNC/collagen			
BNC/silk fibroin			
BNC/Chitosan			
BNC/collagen			
Poly(oligoethylene glycol methacrylate)/CNCs	Injectable hydrogel	Tissue engineering	150
CNFs/gelatin	Cryogels hydrogels	Controlled drug delivery	151–155
CNFs-Ag/alginate		Antibacterial	
BNC/acrylic acid		Drug delivery	
BNC/gelatin			
CNCs/chitosan			

### 5.3. Water purification

With increasing populations and industrialization, the removal of heavy metal ions has become a challenging task for human society today. Conventional methods such as filtration, chemical precipitation, electrochemical treatment, ion exchange, membrane technologies, evaporation and adsorption of activated carbon, *etc.*, are used for removal of toxic ions from wastewater. However, these methods have some disadvantages such as limited range, expensive technology, and energy intensive.^156^ These limitations can be overcome by biosorption which has the potential to remove the metal ions in solution from ppm (parts per million) to ppb (parts per billion) level.^157–159^

Cellulose-based adsorbent materials have drawn significant attention due to their hydrophilicity, and functionalization ability, and easy to tune the different properties such as surface area, aspect ratio, as well as chemical accessibility.^160^ However, conventional cellulose-based adsorbent has limited selectivity and lower adsorption kinetics due to the limited active sites or surface area and difficult to recover from wastewater. In contrast to micrometer-sized cellulose, the nanometer-sized cellulose has a larger surface area with improved porosity, which provides a great advantage for metal ion removal from wastewater. This improvement in the adsorbent potential of nanocellulose is due to the presence of functional groups, increased surface area, and crystalline nature.^161^ In addition, nanocellulose is thermodynamically stable as compared to metallic nanoparticles, which make them a suitable material for this application.^162–164^ The processing of nanocellulose-based membranes for optimal access to functional sites, with high flux and mechanical stability, is a challenging task in water purification. In generals, filling of electrospun mats with nanocellulose; vacuum filtration and coating; and freeze-drying are the most relevant and used techniques for the processing of nanocellulose-based membranes.^165^

Surface hydroxyl groups play an important role in the binding and removal of foreign substances from aqueous medium. Here, we describe the water purification capacity of nanocellulose-based membranes through a few examples. The selected examples demonstrate the very high water purification efficiency (∼98%) of nanocellulose at a very low content in polymer matrices. The higher dye and metal ion-removal efficiency of nanohybrids, make them suitable membrane materials for water purification. Karim *et al.* prepared bio-based hybrid membranes of chitosan/CNC through freeze-drying, followed by a compacting process for the removal of dyes from water. These fabricated membranes successfully removed 98%, 84%, and 70% of the positively charged dyes Victoria Blue 2B, Methyl Violet 2B, and Rhodamine 6G, respectively, after a contact time of 24 h. This activity of the fabricated membranes was attributed to the electrostatic interactions between the positively charged dyes and negatively charged CNC.^166^Fig. 10 presents a naked-eye visualization of the removal efficiency of cross-linked hybrid membranes. A thin-film nanocomposite (TFN) membrane was synthesized through the *in situ* interfacial polymerization of MPD and trimesoyl chloride (TMC) by incorporating CNCs into the active polyamide layer for water desalination. A salt rejection of 97.8% was observed when 0.1% (w/v) of nanocellulose was loaded into the matrix, indicating the potential for the large-scale use of these nanohybrid membranes in water desalination.^167^ Tato *et al.* synthesized thin-film composite (TFC) membranes using amino-modified nanocellulose with silver and platinum nanoparticles. The fabricated thin films showed finger-like pore morphologies and varying pore sizes, and a high solute rejection of wastewater samples, which indicates their potential as novel water purification materials.^168^ In another study, fully bio-based TFCs were synthesized using cellulose microfibers as a support layer and different CNC concentrations in a gelatin matrix as the functional layer. When treated with mirror industry effluents containing metal ions (Ag^+^, Cu^2+^, and Fe^2+^/Fe^3+^), the fabricated film showed a high ion-removal capacity (100% in some cases) and a very high water permeability (900–4000 L h^−1^ m^−2^) at pressure below 1.5 bars. The high ion-removal capacity of these membranes is due to the high level of interactions between positively charged metal ions and the negatively charged CNCs.^169^ The nanocellulose-based polymer hybrids used in water purification are also given in Table 7.

**Table 7:** Nanocellulose-based materials for application of wastewater treatment

Materials	Removal of	Adsorption potential (mg g^−1^)	References
Calcium hydroxyapatite/microfibrillated cellulose (MFC)	Cr(vi)	114.7	170
Aminopropyltriethoxysilane modified (APS)/MFC	Ni(ii)	159.8	171
	Cu(ii)	200.1	
	Cd(ii)	471.5	
Nanocellulose/nanobentonite composite	Co(ii)	350.8	172
	U(vi)	121.0	
Cellulose nanofibers (CNF) with positively charged quaternary ammonium groups	NO_3_^−^	44.0	173
	F^−^	10.6	
	SO_4_^2−^	50.0	
	PO_4_^3−^	55.0	
Cationic CNF aerogel with trimethylammonium chloride	Blue dye CR 19	230.0	174
	Red dye 180	160.0	
	Orange dye 142	230.0	
Amino-modified nanocrystalline cellulose (ANCC)	Acid red GR,	134.7	175
	Congo red 4BS	199.5	
	Light yellow K-4G	183.0	
Carboxylate-modified cellulose nanocrystal (CNCs)	Crystal violet	243.9	176
	Methylene blue		
	Malachite green		
	Basic fuchsin		
CNCs/alginate hydrogel beads	Methylene blue	255.5	177
Phosphorylated nanocellulose	Ag(i)	∼100%	178
	Cu(ii)		
	Fe(iii)		

## 6. Conclusions and future perspectives

Nanocellulose has drawn a lot of attention from the scientific community in recent year, especially in the form of bacterial cellulose, cellulose nanofibrils, and cellulose nanocrystals, owing to their unique inheritance properties, easy fabrication, and biodegradability and biocompatibility. Nanocellulose can be obtained from a variety of natural sources and its properties can be optimized according to the desired application. Nanocellulose is frequently used as a filler material to improve the native properties of polymers. The structural, morphological, thermal, and biological properties of nanohybrids are highly influenced by the type of nanocellulose used. An improvement in the mechanical, thermal, and optical properties was observed in nanohybrids due to the different types of interactions between the polymer matrix and the nanocellulose. Nanocellulose-based hybrids exhibited a sustained drug-release ability and biocompatible nature, indicating their suitability as materials for tissue engineering applications. The removal of toxic metal ions through hybrid films provides a new platform for the use of adsorbent materials. In conclusion, the high aspect ratio and excellent mechanical strength and optical properties of nanocellulose have opened a number of new application fields. The versatile nature of nanocellulose functionalization opens up a new class of biocompatible, biodegradable, and sustainable material for a variety of applications. Numerous studies utilizing the excellent physio-chemical properties of nanocellulose have been published in recent years indicating that nanocellulose-based polymer hybrids will be widely used in medical, biotechnology, and food industries, to name a few*.* We hope that ongoing research on nanocellulose-based polymer hybrids will provide high-quality materials for various applications. This review provides a concise contribution of nanocellulose in the fields of tissue engineering and wastewater purification.

## Conflicts of interest

The authors declare no competing financial interests.

## Abbreviations

BNC: Bacterial nanocellulose
CNFs: Cellulose nanofibrils
CNCs: Cellulose nanocrystals
MCC: Commercial microcrystalline cellulose
MES: 2-(*N*-Morpholino)ethanesulfonic acid
NHS: *N*-Hydroxysuccinimide

## Supplementary Material
